# Intercropping of *Saccharum* spp. with *Dictyophora indusiata*: effects on microbial communities and metabolite profiles during bagasse degradation

**DOI:** 10.3389/fmicb.2025.1510904

**Published:** 2025-01-22

**Authors:** Mingzheng Duan, Xiaojian Wu, Shengfeng Long, Hairong Huang, Xiang Li, Yijie Li, Changning Li, Bin Feng, Jiafu Chen, Defa Zhong, Zhendong Chen, Zeping Wang

**Affiliations:** ^1^Guangxi Academy of Agricultural Sciences, Nanning, China; ^2^Key Laboratory of Edible Fungi Resources Innovation Utilization and Cultivation, College of Agronomy and Life Sciences, Zhaotong University, Zhaotong, China; ^3^Laibin Academy of Agricultural Sciences, Laibin, China

**Keywords:** sugarcane, bagasse, *Dictyophora indusiata*, metabarcoding, metabolomics

## Abstract

**Background:**

Intercropping *Saccharum* spp. (sugarcane) with the fungus *Dictyophora indusiata* together with bagasse amendment represents an innovative circular agriculture method that can enhance soil health, boost sugarcane yields, and increase farm profitability. Understanding the process by which *D. indusiata* degrades bagasse is key to optimizing this method.

**Aims:**

This study aims to clarify the microbial and metabolic processes involved in bagasse degradation by *D. indusiata* in the sugarcane intercropping system.

**Methods:**

Chemical composition analysis, metabarcoding sequencing, and metabolomic profiling were conducted on *D. indusiata*-degraded bagasse (DIBA) and naturally degraded bagasse (BA).

**Results:**

Analysis of chemical composition revealed that only acid detergent fiber (ADF) and crude protein content differed significantly between the DIBA and BA treatments. Metabarcoding sequencing showed that DIBA significantly altered the bacterial and fungal communities, reducing microbial diversity. Metabolomic analysis indicated an enhancement of biological metabolism, particularly carbohydrate breakdown, in the DIBA treatment. Key metabolites, such as glucose, cellobiose, and D-mannose, were more abundant in DIBA samples. In addition, unique metabolites such as L-alanine, serine, and oxaloacetate were detected in the DIBA treatment, suggesting more efficient bagasse degradation compared with natural processes.

**Conclusion:**

The use of macrofungi such as *D. indusiata* can play a pivotal role in circular agriculture by transforming agricultural waste into valuable soil amendments. Future research should focus on the long-term impact of this system on soil quality and crop yield, as well as the underlying mechanisms, to further optimize intercropping systems and the use of fungi in agricultural waste management.

## 1 Introduction

The sustainable production of food has long been an important goal for mankind, but this goal is increasingly challenged by the unabated growth of the human population and the intensification of climate change (Zhang et al., 2018). Large quantities of fertilizer have been applied to accommodate the growing demand for food. However, the excessive application of fertilizer has caused deleterious effects on both crop production (for example, decreased yields) and the environment (such as environmental pollution and reductions in soil biodiversity) in China as well as in several other developing countries (Xu et al., 2020; Cui et al., 2020; Li et al., 2019; Vitousek et al., 2009). There is thus a pressing need to develop approaches that provide some of the beneficial effects of fertilizer application without compromising agricultural productivity or damaging the soil environment (Duan et al., 2022). Bagasse is a plant residue that remains after the extraction of sugar from crops such as sugarcane, and inorganic nutrients [e.g., phosphorus (P) and potassium (K)] required for plant growth are abundant in bagasse (Sharma et al., 2015). It is therefore often used as an auxiliary fertilizer in sugarcane fields after harvest and sugar extraction (Seleiman and Kheir, 2018). The application of bagasse is considered an effective strategy for alleviating the deleterious effects of excessive fertilizer application and more generally for achieving the goals of ecological agriculture, which aims to enhance production while promoting ecological sustainability. Bagasse application has been shown to enhance soil health by increasing the abundance of various soil nutrients (Sharma et al., 2015; Seleiman and Kheir, 2018; Bento et al., 2019) and pH (Mohamed et al., 2019). Moreover, bagasse application promotes the activity of soil microbes and increases the diversity of soil microbial communities (Ullah et al., 2020; Bhadha et al., 2020).

*Dictyophora indusiata* (DI), which is in the family Phallaceae, is an economically valuable, edible mushroom that is used in Chinese cuisine; it is also widely used as a Chinese medicinal material (Wang et al., 2021). Several culture substrates can be used to grow DI and improve soil nutrients, including sugarcane bagasse (Tong et al., 2021). Thus, the application of bagasse to sugarcane fields can enhance sugarcane production and promote soil health.

Building on this, a previous study conducted an intercropping experiment with *D. indusiata* and sugarcane using bagasse as a substrate. The findings revealed that growing *D. indusiata* in bagasse, intended as a fertilizer, not only reduced soil nitrogen loss by inhibiting soil urease synthesis (Duan et al., 2023) but also promoted the formation of a “white root” symbiosis with sugarcane, increasing flavonoid accumulation in the roots and significantly boosting sugarcane yield (Duan et al., 2024). These results suggest that degradation of bagasse by *D. indusiata* offers more soil and crop benefits than does natural degradation. Understanding the bagasse degradation process is crucial for optimizing this intercropping method and advancing circular agricultural techniques. However, the specifics of how *D. indusiata* degrades bagasse during field cultivation remain unclear, limiting further development of this ecological cultivation method.

Macrofungi play a crucial role in the degradation of bagasse and other organic materials (Ahmed et al., 2018). First, they secrete a variety of enzymes, such as cellulases, hemicellulases, and ligninases (Al-Dhabi et al., 2021). These enzymes break down the complex components of bagasse, including cellulose, hemicellulose, and lignin. For example, cellulases hydrolyze cellulose into smaller sugar molecules, which can then be used by the fungus and other microorganisms (Wilson, 2011; Lynd Lee et al., 2002). Hemicellulases act on hemicellulose to release various sugars and other compounds (Saha, 2003). Ligninases degrade lignin, a complex polymer that is difficult to decompose (Laca et al., 2019). As the degradation process proceeds, macrofungi and associated microorganisms consume the degraded products for their growth and metabolism (Lu et al., 2020). This leads to the release of nutrients such as nitrogen, phosphorus, and potassium, which can be absorbed by plants and contribute to soil fertility. In addition, the degradation process can also modify the physical and chemical properties of bagasse, making it more accessible to other microorganisms and promoting the overall activity of the soil microbial community (Liu et al., 2023). Understanding the process by which large fungi degrade bagasse can provide valuable insights for improving intercropping methods and promoting the development of sustainable sugarcane cultivation techniques. By optimizing the degradation process, we can enhance the efficiency of nutrient release from bagasse, improve soil health, and increase crop productivity.

Understanding the microbial diversity and metabolic processes involved in bagasse degradation by *D. indusiata* is key to optimizing this process. Recent advances in second-generation sequencing technologies, particularly metabarcoding sequencing, have enabled the detailed analysis of microbial diversity in macro environments (Duan and Bau, 2021a; Duan and Bau, 2021b; Duan et al., 2023). In addition, derivatization-based metabolomic analysis using Gas Chromatography - Tandem Mass Spectrometry (GC–MS/MS) has emerged as a powerful tool for studying complex metabolites (Duan et al., 2022; Duan et al., 2023). Therefore, combining metabarcoding sequencing and GC–MS/MS-based metabolomics holds promise for revealing the characteristics of *D. indusiata*-mediated bagasse degradation.

This study aimed to clarify the characteristics of bagasse degraded by *D. indusiata* in a sugarcane intercropping system. By analyzing the chemical composition, fungal and bacterial diversity, and metabolomic profiles of bagasse samples cultivated with or without *D. indusiata*, we aimed to provide insights into this degradation process. Notably, our approach integrating multiple advanced techniques to dissect the complex interactions between the macrofungus, microbial communities, and metabolites during bagasse degradation is unprecedented, which sets a new benchmark for future research in this area and offers a novel perspective to optimize circular agricultural practices.

## 2 Materials and methods

### 2.1 Materials

**Figure 1:**
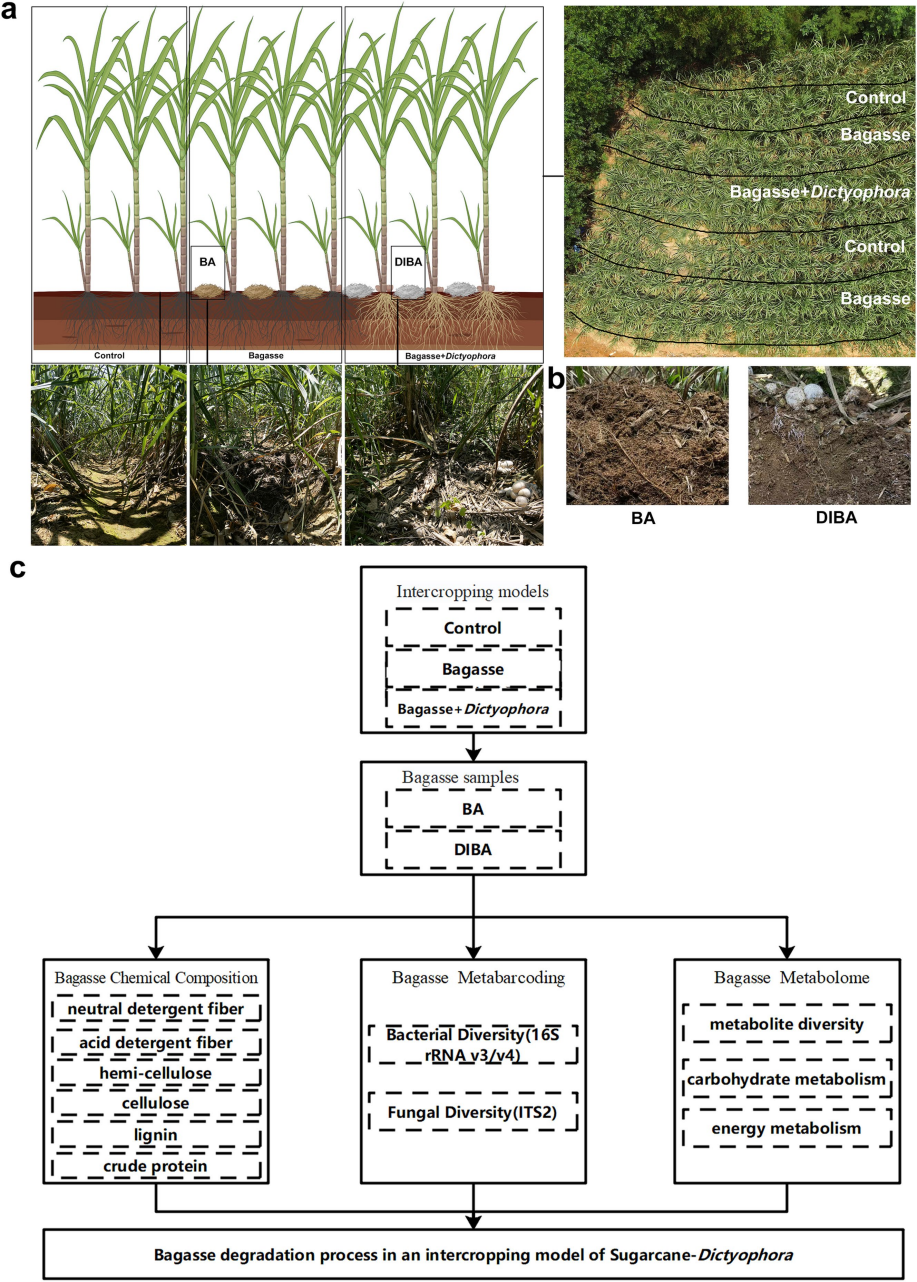
The experimental approach used in this study. **(A)** Sugarcane and *Dictyophora indusiata* intercropping. The image in the upper right shows an aerial view of the field and the division of treatments within the field. The three images in the lower left show sugarcane rows from the different treatments [Control, naturally degraded bagasse (BA) and *D. indusiata*-degraded bagasse (DIBA)]. **(B)** Appearance of the bagasse in the BA and DIBA treatments. **(C)** Flowchart of analyses performed in this study.

The experiment was performed in a field in Laibin City, Guangxi, China (23°16′N, 108°24′E) from February to August 2022. Two treatments were applied: (1) Bagasse process, in which crushed, air-dried bagasse was added to the soil between the sugarcane rows; and (2) Bagasse + *Dictyophora* process, in which crushed, air-dried bagasse mixed with *D. indusiata* strains (variety: Gutian-1; Duan et al., 2023; obtained via wheat-grain fermentation with 35 d) was applied between the rows, all treatments were maintained at high soil moisture (Figure 1A). The locally sourced sugarcane variety GT42 was used throughout the experiment, planted with an interrow spacing of approximately 50 cm and a row depth of 30 cm. The detailed intercropping model settings are consistent with those in the research published in the early stage of this study (Duan et al., 2023).

After 60 days of cultivation, when the sugarcane plants had reached about 50 cm in height, five semi-rectangular areas were marked for the treatments. The control and bagasse processes were each duplicated, whereas the Bagasse + *Dictyophora* process was performed in a single area at the center of the site (Figure 1A). The fruiting bodies of *D. indusiata* matured after roughly 60 days, and soil moisture was maintained at a high level during this period.

As shown in Figures 1A,B, five random points were selected in both the adjacent naturally degraded bagasse (BA) and *D. indusiata*-degraded bagasse (DIBA) areas as biological replicates, as Figure 1 Shown. Bagasse samples were collected by removing the top layer and sampling, and avoid mixing soil and root material, at a depth of 2–10 cm (for each point in the Figure 1A, bagasse from five separate sub-points was mixed for each sample, i.e., totaling 25 samples per treatment). All samples were frozen, ground into powder, packed into sterile cryostorage tubes, and preserved in liquid nitrogen.

### 2.2 Methods

#### 2.2.1 Chemical composition analysis of bagasse

After air-drying, the bagasse samples were crushed. The contents of plant cellulose, hemicellulose, lignin, acid detergent fiber (ADF), and neutral detergent fiber (NDF) were determined using the paradigm washing method combined with a crude fiber analyzer as described previously (Kong et al., 2017). The plant crude protein content was measured using sulfuric acid–catalyst digestion followed by the Kjeldahl nitrogen method (Shang et al., 2022).

#### 2.2.2 Metabarcoding analysis

To extract DNA from bagasse samples (3 g), we used the HiPure Soil DNA Kit (Magen, Guangzhou, China) according to the manufacturer’s protocol as described previously (Duan et al., 2022). The 16S rDNA V3–V4 region of the ribosomal RNA gene was amplified by polymerase chain reaction (PCR) using the primers 341F (5′-CCTACGGGNGGCWGCAG-3′) and 806R (5′-GGACTACHVGGGTATCTAAT-3′) for bacteria (Guo et al., 2017) and the primers ITS3_KYO2 (5′-GATGAAGAACGYAGYRAA-3′) and ITS4 (5′-TCCTCCGCTTATTGATATGC-3′) for fungi (Duan et al., 2022). Sequencing was performed on the Illumina NovaSeq 6,000 platform and involved paired-end sequencing of pooled purified metabarcoding libraries in equimolar ratios according to standard protocols (These steps were performed by Gene Denovo Co., Ltd., Guangzhou, China). Classification of sequences into representative operational taxonomic units (OTUs) was performed using a naïve Bayesian model with The Ribosomal Database Project classifier (version 2.2) (Wang et al., 2007). Annotation was based on the SILVA (16S rRNA metabarcoding data) (Pruesse et al., 2007) and UNITE (ITS metabarcoding data) (Nilsson et al., 2019) (version 8.0) databases. All figures were generated using R software, with the *VennDiagram* package (version 1.6.16) used to compare OTUs among groups (Chen and Boutros, 2011). The Shannon index and other alpha diversity indices were calculated using QIIME (version 1.9.1) (Caporaso et al., 2010). Principal component analysis (PCA) was performed to examine variations in OTU composition among experimental groups using the *vegan* R package.^1^ Statistically significant differences between treatments were determined using Welch’s *t*-test performed with the *vegan* package. The FAPROTAX database (Functional Annotation of Prokaryotic Taxa; version 1.0) (Louca et al., 2016) was used to predict ecologically relevant bacterial functions, and FUNGuild (Nguyen et al., 2016) (version 1.0) was used to predict the functional groups (guilds) of fungi.

#### 2.2.3 Metabolome analysis

Metabolite analysis was performed using a previously published method [6; 15]. Approximately 0.5 g of bagasse sample was mixed with 1 mL of methanol: isopropanol: water (3: 3: 2 v/v/v), vortexed for 3 min, and subjected to ultrasound for 20 min. After centrifugation at 12,000 r/min and 4°C for 3 min, the supernatant was combined with 0.020 mL internal standard (10 μg/mL) in a sample vial and evaporated under nitrogen. The residue was freeze-dried in a lyophilizer, derivatized by mixing with 0.1 mL methoxy-amine hydrochloride in pyridine (0.015 g/mL), and incubated at 37°C for 2 h. Then, 0.1 mL bis (trimethylsilyl) trifluoroacetamide (BSTFA) [with 1% trimethylchlorosilane (TMCS)] was added, and the mixture was kept at 37°C for 30 min after vortexing. Approximately 0.2 mL of the derivatization solution was diluted with n-hexane to 1 mL, filtered through a 0.22-μm organic phase syringe filter, stored at −20°C, and analyzed within 24 h.

Metabolites were analyzed by gas chromatography–mass spectrometry (GC–MS) on an Agilent 8,890 gas chromatograph coupled to a 5977B mass spectrometer with a DB-5MS column (30 m length × 0.25 mm i.d. × 0.25 μm film thickness, J&W Scientific, United States). Helium was used as the carrier gas at a 1.2 mL/min flow rate. Samples were injected in front inlet mode with a split ratio of 5:1, and the injection volume was 1 μL. The oven temperature protocol consisted of an initial hold at 40°C for 1 min, ramping to 100°C at 20°C/min, further ramping to 300°C at 15°C/min, and a final hold at 300°C for 5 min. All samples were scanned in scan mode with ion source and transfer line temperatures of 230°C and 280°C, respectively.

Statistical analysis involved unsupervised PCA using the *prcomp* function in R, with data scaled to unit variance prior to PCA. Metabolites with a variable importance in projection (VIP) ≥ 1 and absolute log2 (fold change) (log2FC) ≥ 1 were considered to differ significantly between the BA and DIBA treatments. VIP values were obtained from orthogonal partial least squares discriminant analysis (OPLS-DA) results, which included score plots and permutation plots generated with the R package *Metabo-AnalystR*. Prior to OPLS-DA, the original data were log2-transformed and mean-centered, and a permutation test (200 permutations) was performed to prevent overfitting.

## 3 Results

### 3.1 Chemical composition of bagasse

**Figure 2:**
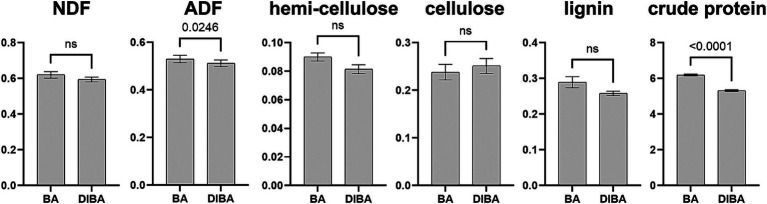
Chemical composition of bagasse from the BA and DIBA treatments (*n* = 3). The vertical axis units are percentage for neutral detergent fiber (NDF), acid detergent fiber (ADF), hemicellulose, cellulose, and lignin and g/100 g for crude protein. Data are expressed as mean ± SD. Numbers above the bars indicate the significance of differences between the two treatments based on Welch’s *t*-test. ns, *p* > 0.05. NDF, neutral detergent fiber. ADF, acid detergent fiber.

We first analyzed differences in the chemical composition of bagasse from the BA and DIBA treatments. As shown in Figure 2, among the six measured indicators, only ADF (with mean values of 52.99% for BA and 51.2% for DIBA) and crude protein (with mean values of 6.18 g/100 g for BA and 5.31 g/100 g for DIBA) showed significant differences (*p* < 0.05). We concluded that DI degradation and natural degradation had relatively similar effects on the overall chemical composition of bagasse.

### 3.2 Bagasse bacterial and fungal diversity

#### 3.2.1 OTU distribution and alpha diversity

**Figure 3:**
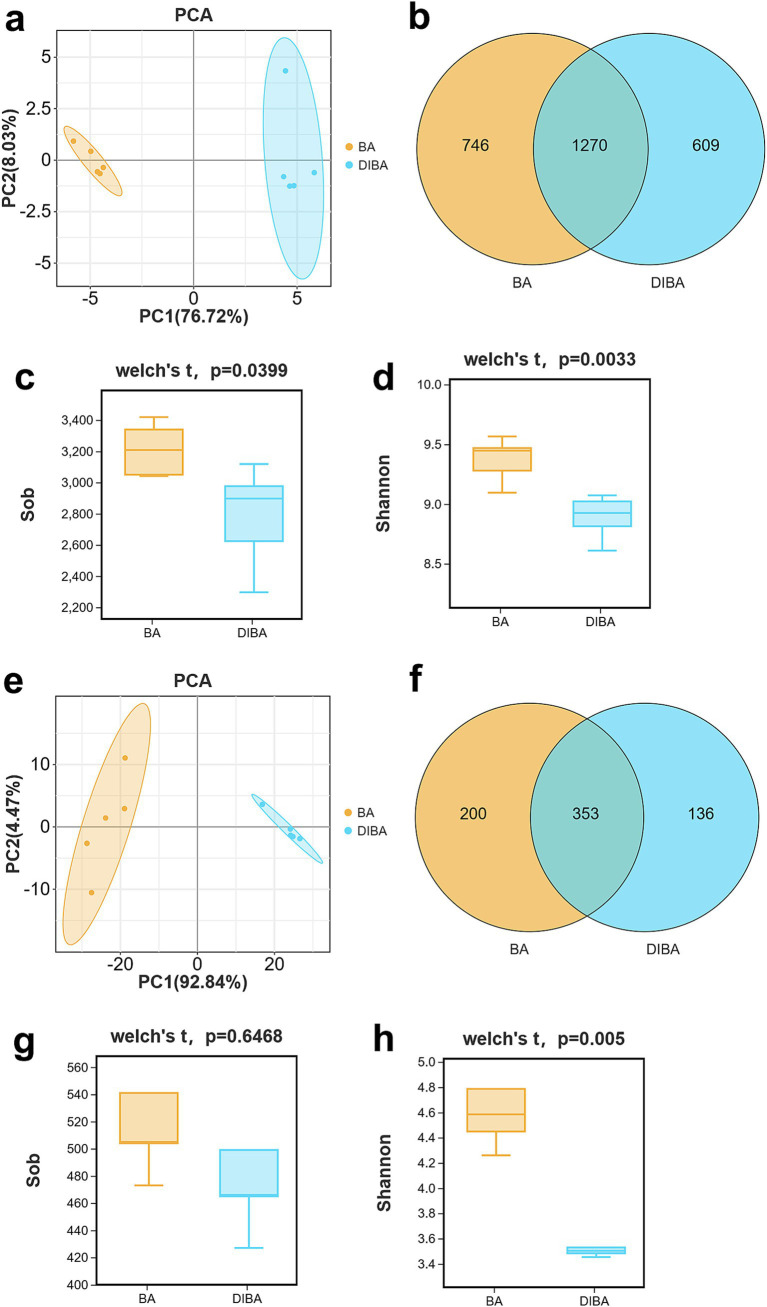
OTUs and alpha diversity of bacteria and fungi in bagasse from the BA and DIBA treatments (*n* = 5). **(A)** Principal Component Analysis (PCA) plot of bacterial OTUs. **(B)** Venn diagram of bacterial OTUs. **(C)** Observed species (Sob) index of bacteria in the naturally degraded bagasse (BA) and *D. indusiata*-degraded bagasse (DIBA) treatments. **(D)** Shannon index of bacteria in the BA and DIBA treatments. **(E)** PCA plot of fungal OTUs. **(F)** Venn diagram of fungal OTUs. **(G)** Sob index of fungi in the BA and DIBA treatments. **(H)** Shannon index of fungi in the BA and DIBA treatments. In all boxplots, the top and bottom whiskers indicate the maximum and minimum values, the top margin of the box indicates the upper quartile, the lower margin indicates the lower quartile, and the central line represents the mean.

To explore the microbial processes involved in DI degradation of bagasse, we performed metabarcoding sequencing of replicated bagasse samples from the BA and DIBA treatments, targeting 16S rRNA for bacteria and ITS for fungi. The sequencing generated 2,086,246 tags, with an average N50 length of 447 bp for bacteria and 361 bp for fungi. The range of OTUs across biological replicates (*n* = 5) was 2,296–3,419 for bacteria (mean 2,996) and 427–640 for fungi (mean 519), as detailed in Supplementary Tables S1, S2. PCA plots showed clear clustering of BA and DIBA samples from both the bacterial and fungal communities, with bacterial resolution (Figure 2A, PC1 76.72% and PC2 8.03%) and fungal resolution (Figure 2E, PC1 92.84% and PC2 4.47%) both exceeding 75%, indicating high separation accuracy. Venn diagrams of the OTUs revealed a total of 2,625 bacterial OTUs (746 unique to BA, 609 unique to DIBA, and 1,270 shared) and 689 fungal OTUs (200 unique to BA, 136 unique to DIBA, and 353 shared; Figures 3B,F). Alpha diversity analysis (Supplementary Table S3) showed that the observed species (Sob) index (Figure 3C) and the Shannon index (Figure 3D) for bacteria were significantly higher in BA (means 3,211 and 9.37) than in DIBA (means 2,782 and 8.88; *p* < 0.05), indicating that bacterial diversity was higher in BA. For fungi Shannon index (Supplementary Table S4), there was no significant difference in the Sob index between the BA (mean 532) and DIBA (mean 507) treatments, but the Shannon index was significantly higher in the BA treatment (mean 4.69) than in the DIBA treatment (mean 3.67) (*p* < 0.05; Figure 3G). Overall, these results suggest that DI degradation of bagasse significantly altered both bacterial and fungal diversity, leading to a decrease in alpha diversity.

#### 3.2.2 Taxonomy and functions of bacteria and fungi in bagasse

**Figure 4:**
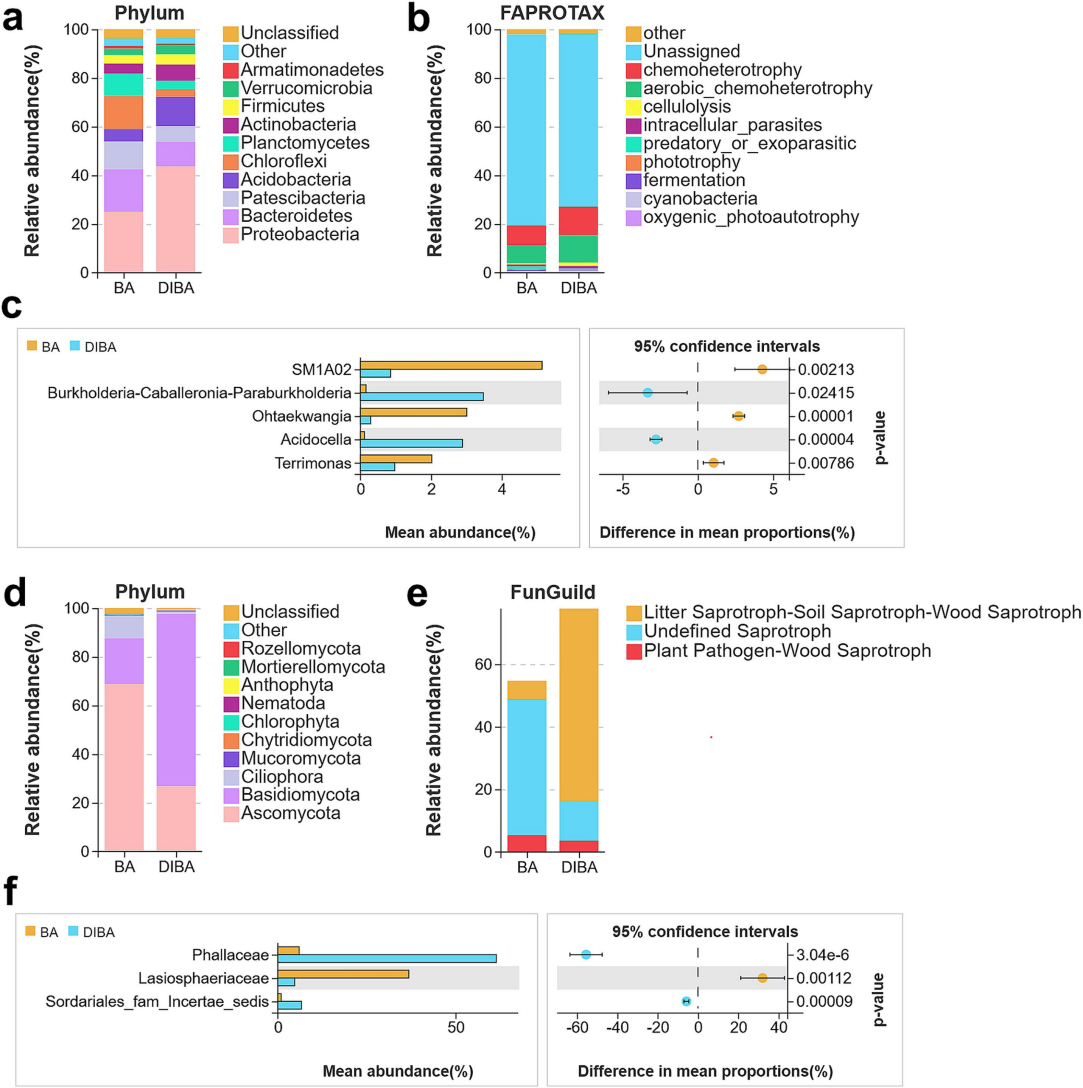
Bacterial and fungal taxa and functions in bagasse from the BA and DIBA treatments. **(A)** Relative abundance of bacterial phyla in naturally degraded bagasse (BA) and *D. indusiata*-degraded bagasse (DIBA) samples. **(B)** Functional annotations of bacterial OTUs obtained from Functional Annotation of Prokaryotic Taxa (FAPROTAX) database. **(C)** The five bacterial taxa with the greatest difference in abundance between BA and DIBA treatments. Significant differences in mean abundance were assessed using Welch’s *t*-test. **(D)** Relative abundance of fungal phyla in BA and DIBA samples. **(E)** Functional annotations of fungal OTUs obtained from the FunGuild database. **(F)** The three fungal taxa with the greatest difference in abundance between BA and DIBA treatments. Significant differences in mean abundance were assessed using Welch’s *t*-test.

Next, we examined the most abundant taxa of bacteria and fungi present in bagasse from the BA and DIBA treatments. The top five most abundant bacterial phyla (Figure 4A) were Proteobacteria (25.04% in BA, 43.59% in DIBA), Bacteroidetes (17.51, 10.16%), Patescibacteria (11.26, 10.16%), Acidobacteria (5.18, 12.13%), and Chloroflexi (13.53, 3.03%). The top three most abundant fungal phyla (Figure 4D) were Ascomycota (68.71% in BA, 26.77% in DIBA), Basidiomycota (18.92, 70.86%), and Ciliophora (9.26, 0.85%). When bacteria in the BA and DIBA treatments were compared, the five bacterial genera with the most significant differences in relative abundance (Figure 4C) were SM1A02 (5.14% in BA, 0.85% in DIBA), Burkholderia-Caballeronia-Paraburkholderia (0.15, 3.47%), Ohtaekwangia (3, 0.28%), Acidocella (0.11, 2.88%), and Terrimonas (2.02, 0.96%). Likewise, the three fungal families (Figure 4F), with the greatest differences in relative abundance were Phallaceae (5.97% in BA, 61.46% in DIBA), Lasiosphaeriaceae (36.84, 4.73%), and Sordariales_fam_Incertae_sedis (0.92, 6.61%).

Finally, we examined the functional roles of the identified microorganisms. Using the FAPROTAX database (Figure 4B), we annotated bacterial OTUs and found that the three functional modules with the largest differences in abundance between BA and DIBA samples were chemoheterotrophy (8.11% in BA, 11.66% in DIBA), aerobic chemoheterotrophy (7.5, 11.2%), and cellulolysis (0.52, 1.43%). An analysis of fungal OTUs, annotated using the FunGuild database (Figure 4E), revealed similar functional modules, with the largest differences also observed in chemoheterotrophy, aerobic chemoheterotrophy, and cellulolysis.

### 3.3 Soil metabolome

#### 3.3.1 Composition of bagasse metabolites

We next performed a metabolomic analysis to further characterize the process of bagasse degradation by DI. A total of 100 metabolites were detected, including acids (22), alcohols (12), amines (3), aromatics (3), carbohydrates (22), esters (4), heterocyclic compounds (4), ketones (1), lipids (18), nitrogen compounds (1), phenols (1), and others (9).

**Figure 5:**
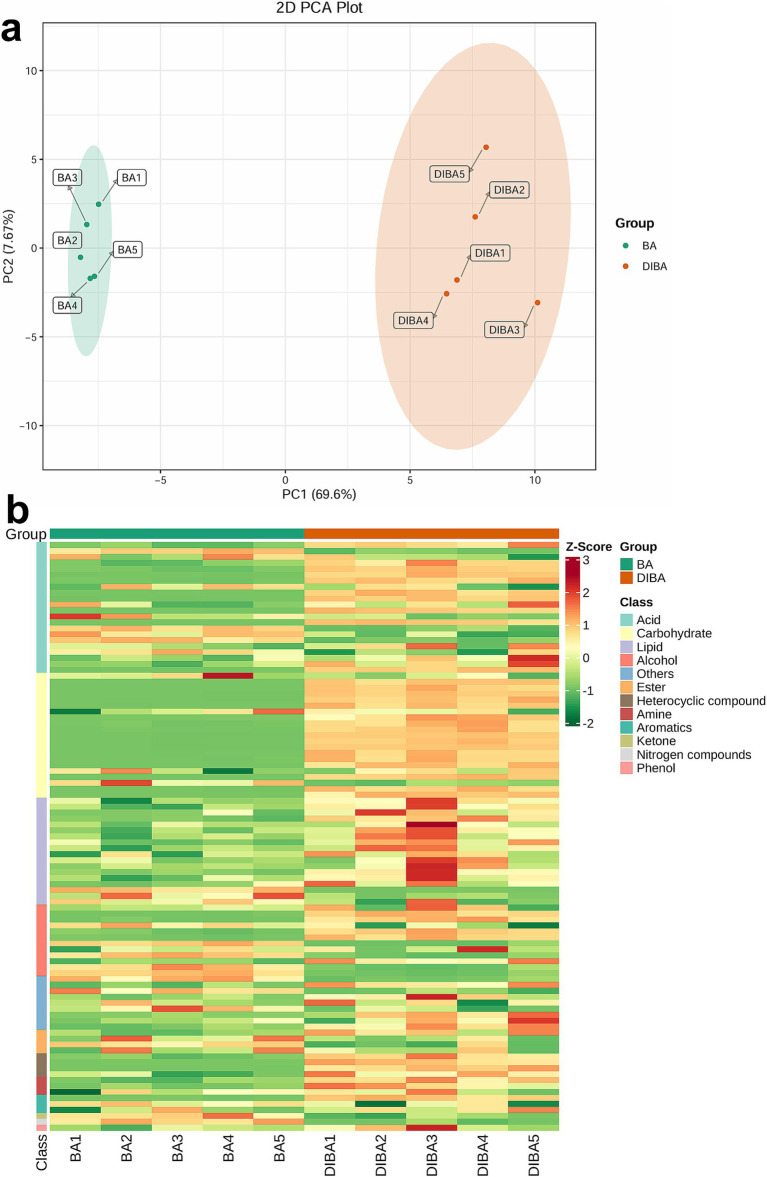
Composition and abundance of metabolites in BA and DIBA samples. **(A)** Principal Component Analysis (PCA) plot of metabolite abundance in naturally degraded bagasse (BA) and *D. indusiata*-degraded bagasse (DIBA) samples. **(B)** Heatmap showing the *Z*-score-normalized abundance values of individual metabolites from 12 classes in BA and DIBA samples. Each column corresponds to a sample, and each row corresponds to a metabolite.

We first analyzed the overall patterns of metabolite abundance in BA and DIBA samples using PCA. As shown in Figure 5A, PC1 (69.6%) and PC2 (7.67%) together explained 77.27% of the variance, and the treatments were clearly separated along PC1. Most of the identified metabolites, including those from key categories such as acids, carbohydrates, and lipids, had higher abundances in DIBA samples than in BA samples (Figure 5B). For detailed information on all metabolites and their relative abundances, please refer to Supplementary Table S5. These findings suggest that the addition of DI to bagasse-amended soil significantly may increase the intensity of biological metabolism, accelerating bagasse degradation.

#### 3.3.2 Identification of differentially abundant metabolites

We identified metabolites whose abundance differed significantly between the BA and DIBA treatments. As shown in Tables 1, 46 of the 100 detected metabolites exhibited significant differences in abundance between the BA and DIBA samples. The three metabolites with the greatest increase in abundance in DIBA compared with BA were 4-aminobutanoic acid (Log2FC = 15.7), *β*-gentiobiose octamethyl (Log2FC = 11.75), and D-galactose 2 (Log2FC = 9.27). Notably, 17 of the 22 detected carbohydrate metabolites (77.27%) were significantly more abundant in DIBA, suggesting that carbohydrate and energy metabolism were particularly stimulated during the process of bagasse degradation by DI.

**Table 1:** Forty-six differentially abundant metabolites between the naturally degraded bagasse (BA) and *D. indusiata*-degraded bagasse (DIBA) treatments.

Type	Formula	Compound	*p*- value	Log2FC
Acid	CH_2_O_3_	Carbonic Acid 1	3.65E−05	1.33
Acid	C_3_H_7_NO_2_	N,N-dimethyl-Carbamic Acid	9.89E−07	−1.60
Acid	C_3_H_6_O_3_	Lactic Acid	1.36E−07	1.84
Acid	C_2_H_4_O_3_	Hydroxy-Acetic Acid	1.28E−05	4.55
Acid	C_4_H_8_O_3_	3-hydroxy-Butanoic Acid	1.02E−05	3.71
Acid	C_4_H_6_O_4_	Butanedioic Acid	2.76E−05	6.37
Acid	C_4_H_4_O_4_	Fumaric Acid	1.83E−06	5.70
Acid	C_4_H_9_NO_2_	4-Aminobutanoic Acid	6.09E−06	15.70
Acid	C_8_H_8_O_4_	Vanillic Acid	7.20E−06	7.18
Acid	C_18_H_34_O_2_	Cis-Vaccenic Acid	5.29E−06	−1.38
Acid	C_24_H_48_O_2_	Tetracosanoic Acid	1.50E−06	2.29
Alcohol	C_3_H_8_O_3_	Glycerin	4.26E−05	2.12
Alcohol	C_6_H_12_O_6_	Myo-Inositol 1	1.59E−04	2.62
Alcohol	C_6_H_12_O_6_	Scyllo-Inositol	1.90E−06	2.22
Alcohol	C_6_H_12_O_6_	Myo-Inositol 3	4.25E−05	3.48
Alcohol	C_18_H_38_O	1-Octadecanol	1.07E−07	−1.27
Alcohol	C_28_H_44_O	Ergosterol	2.82E−06	−1.17
Alcohol	C_29_H_48_O	Stigmasterol 2	6.08E−05	−1.06
Alcohol	C_29_H_50_O	β-Sitosterol 1	7.00E−07	−1.46
Amine	C_5_H_10_N_2_O	2-amino-N-cyclopropylacetamide	3.85E−03	2.08
Aromatics	C_7_H_5_NO	4-hydroxy-Benzonitrile	2.50E−05	3.60
Carbohydrate	C_6_H_12_O_6_	D-Galactose 2	4.96E−06	9.27
Carbohydrate	C_5_H_10_O_5_	d-Arabinose 2	1.77E−05	6.18
Carbohydrate	C_5_H_10_O_5_	Xylulose	7.12E−07	5.58
Carbohydrate	C_5_H_12_O_5_	D-Arabinitol 2	2.14E−05	4.75
Carbohydrate	C_6_H_12_O_5_	Rhamnose	2.97E−05	4.38
Carbohydrate	C_6_H_12_O_6_	D-Allofuranose	6.47E−04	3.87
Carbohydrate	C_6_H_12_O_6_	Fructose 2	1.14E−04	3.86
Carbohydrate	C_6_H_12_O_6_	D-Allose 1	4.35E−05	5.58
Carbohydrate	C_6_H_12_O_6_	D(+)-Talose 1	3.39E−08	5.63
Carbohydrate	C_6_H_12_O_6_	D-Allose 2	3.43E−06	6.56
Carbohydrate	C_6_H_14_O_6_	Sorbitol 1	1.37E−08	5.74
Carbohydrate	C_8_H_15_NO_6_	N-Acetyl-D-glucosamine 1	4.34E−05	5.68
Carbohydrate	C_7_H_14_O_7_	Galacto-heptulose 2	4.80E−05	5.46
Carbohydrate	C_6_H_14_O_6_	D-Mannitol 2	5.54E−05	6.65
Carbohydrate	C_17_H_30_O_12_	4-(2-Methylbutanoyl)Sucrose	7.51E−06	7.99
Carbohydrate	C_12_H_22_O_11_	β-D-galactopyranosyl-4-O-β-D-Glucopyranose 1	3.62E−05	3.51
Carbohydrate	C_20_H_38_O_11_	β-Gentiobiose octamethyl	1.23E−04	11.75
Heterocyclic compound	C_8_H_17_NO_2_	2-(2-Isopropyloxazolidin-3-yl)ethan-1-ol	1.68E−04	1.01
Heterocyclic compound	C_6_H_11_NO_2_	2-Piperidinecarboxylic acid	1.49E−04	8.90
Heterocyclic compound	C_9_H_12_N_2_O_6_	Uridine	1.17E−04	5.51
Lipid	C_13_H_28_	4,6-dimethyl-Undecane	1.09E−04	1.77
Lipid	C_20_H_36_O_2_	9(E),11(E)-Conjugated linoleic acid, ethyl ester	1.28E−04	−3.35
Nitrogen compounds	CH_4_N_2_O_2_	Hydroxyurea	2.22E−05	−1.13
Others	C_13_H_10_N_2_S	Sulfonium, (cyanoamino)diphenyl-, hydroxide, inner salt	5.14E−04	−1.49
Others	C_26_H_44_F_3_N	3-Trifluoromethylbenzylamine, N,N-dinonyl	1.35E−02	1.42

#### 3.3.3 Analysis of carbohydrate metabolites in bagasse from BA and DIBA treatments

To further investigate the metabolites associated with bagasse degradation by DI, we quantified the absolute concentrations of 33 carbohydrates, including 25 monosaccharides, 6 disaccharides, and 1 trisaccharide, in BA and DIBA samples. As shown in Table 2, we detected 17 carbohydrate metabolites, including 4 disaccharides and 13 monosaccharides. The carbohydrate that showed the greatest difference in concentration between BA and DIBA samples was glucose, with concentrations of 27.61 ± 1.27 mg/g in BA samples and 510.04 ± 38.37 mg/g in DIBA samples (Log2FC = 4.21). In addition, cellobiose, D-mannose, and D-galactose were detected only in the DIBA samples. Sucrose was the only carbohydrate whose concentration was higher in BA samples (24.43 ± 1.41 mg/g) than in DIBA samples (6.66 ± 0.25 mg/g; Log2FC = −1.87). These findings suggest that the presence of DI favors the breakdown of disaccharides into monosaccharides for further metabolism, potentially enhancing the efficiency of sugar degradation. Detailed metabolite information is provided in Supplementary Table S6.

**Table 2:** Concentrations of carbohydrate metabolites in naturally degraded bagasse (BA) and *D. indusiata*-degraded bagasse (DIBA) samples (*n* = 5).

Class	Compounds	BA (mg/g)	DIBA (mg/g)	Log2FC	Type
Disaccharide	Cellobiose	0 ± 0	25.42 ± 1.6	/	up
Disaccharide	Trehalose	1907.04 ± 101.25	2166.48 ± 654.48	0.18	ns
Disaccharide	Sucrose	24.43 ± 1.41	6.66 ± 0.25	−1.87	down
Disaccharide	Maltose	7.41 ± 0.37	20.56 ± 1.23	1.47	up
Monosaccharide	1,5-Anhydroglucitol	18.42 ± 1.89	16.65 ± 0.69	−0.15	ns
Monosaccharide	D-Xylulose	2.51 ± 0.18	3.1 ± 0.17	0.30	ns
Monosaccharide	D-Xylose	7.47 ± 0.23	16.62 ± 0.51	1.15	up
Monosaccharide	Xylitol	4.5 ± 0.14	6.64 ± 0.17	0.56	ns
Monosaccharide	L-Rhamnose	7.47 ± 0.21	7.61 ± 0.14	0.03	ns
Monosaccharide	Inositol	10.68 ± 0.23	13.39 ± 0.67	0.33	ns
Monosaccharide	D-Glucuronic acid	6.25 ± 0.13	6.04 ± 0.09	−0.05	ns
Monosaccharide	Glucose	27.61 ± 1.27	510.04 ± 38.37	4.21	up
Monosaccharide	D-Fructose	2.75 ± 0.15	4.6 ± 0.43	0.74	ns
Monosaccharide	D-Arabinose	8.17 ± 0.25	9.47 ± 0.19	0.21	ns
Monosaccharide	D-Arabinitol	6.25 ± 0.49	6.37 ± 0.22	0.03	ns
Monosaccharide	D-Mannose	0 ± 0	12.02 ± 0.44	/	up
Monosaccharide	D-Galactose	0 ± 0	9.2 ± 0.2	/	up

“Type” indicates a significantly higher (up) or lower (down) concentration of the metabolite in DIBA samples compared with BA samples (*p* < 0.05), with ns indicating no significant difference.

#### 3.3.4 Analysis of energy metabolism–related metabolites in bagasse from BA and DIBA treatments

Finally, we quantified the absolute concentrations of 68 energy metabolism–related metabolites in bagasse from the BA and DIBA treatments. As shown in Table 3, we detected 17 metabolites, including amino acid derivatives (1), amino acids (7), nucleotides and their metabolites (5), organic acids and their derivatives (2), and phosphate sugars (1). The three metabolites with the greatest increases in concentration in DIBA compared with BA were threonine (49.95 ± 26.88 ng/g in BA, 2191.56 ± 143.96 ng/g in DIBA; Log2FC = 5.46), glutamine (145.89 ± 81.04 ng/g in BA, 3025.07 ± 246.72 ng/g in DIBA; Log2FC = 5.46), and D(+)-glucose (19717.77 ± 2172.52 ng/g in BA, 383091.1 ± 21446.91 ng/g in DIBA; Log2FC = 4.28). Four additional metabolites—L-asparagine, L-alanine, serine, and oxaloacetate—were detected only in the DIBA samples. Overall, the concentrations of most energy-related metabolites were significantly higher in DIBA samples than in BA samples, indicating that the presence of DI was associated with much higher metabolic intensity during bagasse degradation.

**Table 3:** Energy metabolism–related metabolites in bagasse from the naturally degraded bagasse (BA) and *D. indusiata*-degraded bagasse (DIBA) treatments (*n* = 5).

Class	Compounds	BA (ng/g)	DIBA (ng/g)	Log2FC	Type
Amino acids	Glutamine	145.89 ± 81.04	3025.07 ± 246.72	4.37	up
Amino acids	L-Asparagine	0 ± 0	1183.61 ± 141.91	/	up
Amino acids	L-Alanine	0 ± 0	4398.55 ± 594.58	/	up
Amino acids	L-Leucine	659.19 ± 109.63	2449.64 ± 100.12	1.89	up
Amino acids	Arginine	814.46 ± 73.59	1760.49 ± 117.55	1.11	up
Amino acids	Serine	0 ± 0	2581.03 ± 176.23	/	up
Amino acids	Threonine	49.95 ± 26.88	2191.56 ± 143.96	5.46	up
Amino acids	Tyrosine	267 ± 46.31	3350.08 ± 372.02	3.65	up
Nucleotides and their metabolites	UDP-GlcNAc	0 ± 0	106.82 ± 2.89	/	up
Nucleotides and their metabolites	Uracil	341.35 ± 13.78	132.52 ± 14.85	−1.37	down
Nucleotides and their metabolites	Guanosine	135.41 ± 26.22	1256.88 ± 52.74	3.21	up
Nucleotides and their metabolites	Adenine	371.2 ± 16.08	1999.21 ± 56.36	2.43	up
Nucleotides and their metabolites	Inosine	86.88 ± 11.3	191.52 ± 9.16	1.14	up
Organic acids and their derivatives	Oxaloacetate	0 ± 0	252.21 ± 45	/	up
Organic acids and their derivatives	Succinic Acid	215.95 ± 31.62	383.9 ± 140.55	0.83	ns
Phosphate sugars	D(+)-Glucose	19717.77 ± 2172.52	383091.1 ± 21446.91	4.28	up

“Type” indicates a significantly higher (up) or lower (down) concentration of the metabolite in DIBA samples compared with BA samples (*P* < 0.05), with ns indicating no significant difference.

## 4 Discussion

Interplanting sugarcane with *D. indusiata* is an innovative cultivation method that can improve soil health and sugarcane yield [15; 16]. This study aimed to better understand the consequences of this process for bagasse degradation in order to promote the improvement of this circular agricultural practice. This study examined the degradation of sugarcane bagasse under two conditions: natural degradation (BA) and degradation in the presence of *D. indusiata* (DIBA). Our analyses revealed key chemical, microbial, and metabolite differences between these two treatments, which suggested that DIBA was more effective than BA in terms of bagasse degradation. These findings will be useful for the continued development of agricultural practices based on bagasse application.

Analysis of chemical composition revealed significant differences in ADF and crude protein content between the BA and DIBA treatments, although there were no significant differences in other components such as NDF, hemicellulose, cellulose, and lignin. This result suggests that although DIBA and BA produce similar overall changes in the major structural components of bagasse, DIBA may exert more subtle effects, particularly on protein content. In general, macrofungi are effective in degrading both the NDF and ADF of crop straw. For example, one study showed that use of the fungus *Phanerochete chrysosporium* for biological delignification during fermentation of sugarcane top fiber increased the NDF degradation rate by 54.21% and the ADF degradation rate by 53.06% (Yanti et al., 2021). In addition, related studies have shown that white rot fungi such as *Phanerochaete chrysosporium*, *Irpex lacteus*, and *Phlebia acerina* and edible fungi such as *Pleurotus ostreatus*, *Pleurotus eryngii*, and *Lentinula edodes* can effectively degrade ADF and NDF in straw materials from oats and corn (Zheng et al., 2021; Atalar and Çetinkaya, 2020). Although there was no significant difference in NDF percentage between DIAB and BA samples in our study, the average percentages of both hemicellulose and lignin were lower in DIAB than in BA (Figure 1), consistent with previous reports that macrofungi are effective at degrading ADF and NDF. In addition, the significantly lower crude protein content in DIBA samples suggested that DIBA treatment stimulated the breakdown of proteins, potentially increasing the availability of nitrogen in the soil, as protein is the main source of organic nitrogen in most ecosystems (Jan et al., 2009). In an earlier report, we showed that intercropping with *D. indusiata* reduced nitrogen loss from the soil (Duan et al., 2023). Combining these results, we speculate that increased decomposition of crude protein may be an important aspect of the process by which the DIBA treatment promotes soil health.

Metabarcoding sequencing revealed significant differences in the bacterial and fungal communities of bagasse from the BA and DIBA treatments. DIBA significantly altered microbial diversity, leading to reduced bacterial and fungal alpha diversity compared with BA. This reduction in diversity, particularly the lower Shannon indices, suggests that *D. indusiata* cultivation can reduce microbial diversity during bagasse degradation. Our previous research also found that the diversity of soil and sugarcane root-associated bacteria and fungi decreased when *D. indusiata* was intercropped with sugarcane, consistent with the results of this study. We speculate that this may be because *D. indusiata*, as a dominant fungus, synthesizes antimicrobial metabolites that inhibit the growth of other microorganisms (Huang et al., 2011; Habtemariam, 2019). Notably, the increased abundance of Proteobacteria and Acidobacteria in DIBA, together with the enrichment of Basidiomycota fungi, suggests that DIBA may promote specific microbial taxa that could facilitate the breakdown of complex bagasse materials. Interestingly, our previous study on the diversity of endophytic bacteria in sugarcane roots also found that *D. indusiata* forms a symbiotic relationship with the roots that selectively cultivates specific bacteria (e.g., *Bacillus* spp.) (Duan et al., 2024), again consistent with our current findings. In the fungal community, the increase in Phallaceae and the dominance of Basidiomycota in DIBA underscore the importance of these fungal taxa in lignocellulose degradation. Because Basidiomycota are known for their lignin-degrading capabilities (Lundell et al., 2010), this taxonomic shift in DIBA may have contributed to more efficient degradation of lignocellulosic materials, consistent with the observed higher glucose concentrations in DIBA samples. Given the potential antimicrobial properties of *D. indusiata,* which study had reported that the water extract of *D. indusiate* can inhibit the growth of bacteria and fungi (Habtemariam, 2019), we hypothesize that *D. indusiata* may facilitate the degradation of bagasse by enriching the abundance of specific bacterial and fungal taxa.

Metabolomic analysis suggested that DIBA significantly enhanced biological metabolism, particularly the breakdown of carbohydrates. This was evident from the higher concentrations of key metabolites such as glucose, cellobiose, and D-mannose in DIBA compared with BA. The much higher concentration of glucose in DIBA samples (510.04 pg./g vs. 27.61 pg./g in BA) strongly suggests that the presence of *D. indusiata* promotes the enzymatic hydrolysis of polysaccharides into simpler sugars, which are then metabolized by the microbial community. The metabolism of large fungi typically demands a substantial amount of energy from their environment, resulting in significant activity of carbohydrate-related metabolism. Our previous research also found that carbohydrate metabolism in the soil increased significantly during intercropping of *D. indusiata* with sugarcane (Duan et al., 2023). A study of the interactions between fairy ring fungi and soil also revealed that large fungi promoted a significant increase in soil carbohydrate metabolites (Duan et al., 2022). In addition, the significantly higher concentrations of carbohydrate metabolites in DIBA samples, particularly monosaccharides like D-galactose and cellobiose, support the notion that the DIBA treatment is more effective at breaking down complex carbohydrates into simpler, readily metabolizable forms. This enhanced carbohydrate degradation is further supported by the higher abundance of cellulolytic functional modules in DIBA, as indicated by the FAPROTAX and FunGuild annotations.

Through quantitative metabolomic analysis of carbohydrate and energy metabolites, we identified several metabolites that were unique to the DIBA treatment, including D-mannose, L-alanine, serine, and oxaloacetate. D-mannose is a naturally occurring bioactive monosaccharide, a C-2 epimer of glucose, and a component of various polysaccharides in plants (Wang et al., 2022). L-alanine and serine are key components for protein synthesis, and oxaloacetate is an intermediate in the tricarboxylic acid cycle, which plays important roles in regulating mitochondrial function, gluconeogenesis, the urea cycle, and amino acid synthesis (Yu and Sivitz, 2020). The presence of these unique monosaccharides and amino acid metabolites in DIBA also suggests that DIBA has an enhanced capacity to degrade bagasse compared with BA.

## 5 Conclusion

This study provides evidence that the cultivation of *D. indusiata* alongside sugarcane enhances the degradation of bagasse, suggesting that such intercropping is an effective method for improving soil health and promoting sustainable agriculture. DIBA not only altered the microbial community but also appeared to promote the breakdown of complex carbohydrates, leading to a higher abundance of metabolites essential for soil fertility. These findings suggest that the use of macrofungi like *D. indusiata* could help to advance circular agriculture by converting agricultural waste into valuable soil amendments. Future research should explore the long-term effects of DIBA on soil quality and crop yield, as well as the underlying mechanisms responsible for its selective enrichment of microbial taxa. Understanding these processes could lead to further optimization of intercropping systems and to the more widespread use of fungi in agricultural waste management.

## Data Availability

The original contributions presented in the study are included in the article/Supplementary material, further inquiries can be directed to the corresponding authors.

## Footnotes

^1^version 2.5.3; http://CRAN.R-project.org/package=vegan; 2022.11.9
